# Comparative transcriptomic analysis reveals gene expression associated with cold adaptation in the tea plant *Camellia sinensis*

**DOI:** 10.1186/s12864-019-5988-3

**Published:** 2019-07-31

**Authors:** Yeyun Li, Xuewen Wang, Qiuyan Ban, Xiangxiang Zhu, Changjun Jiang, Chaoling Wei, Jeffrey L. Bennetzen

**Affiliations:** 1 State Key Laboratory of Tea Plant Biology and Utilization/International Joint Laboratory on Tea Chemistry and Health Effects, Anhui Agricultural University, West 130 Changjiang Road, Hefei, 230036 Anhui People’s Republic of China; 2 Department of Genetics, University of Georgia, Athens, USA

**Keywords:** Assamica tea, *Camellia sinensis*, Cold acclimation, Gene expression profiling, Low temperature response

## Abstract

**Background:**

Low temperature restricts the planting range of all crops, but cold acclimation induces adaption to cold stress in many plants. *Camellia sinensis*, a perennial evergreen tree that is the source of tea, is mainly grown in warm areas. *Camellia sinensis var. sinensis* (CSS) has greater cold tolerance than *Camellia sinensis var. assamica* (CSA). To gain deep insight into the molecular mechanisms underlying cold adaptation, we investigated the physiological responses and transcriptome profiles by RNA-Seq in two tea varieties, cold resistant SCZ (classified as CSS) and cold susceptible YH9 (classified as CSA), during cold acclimation.

**Results:**

Under freezing stress, lower relative electrical conductivity and higher chlorophyll fluorescence (Fv/Fm) values were detected in SCZ than in YH9 when subjected to freezing acclimation. During cold treatment, 6072 and 7749 DEGs were observed for SCZ and YH9, respectively. A total of 978 DEGs were common for both SCZ and YH9 during the entire cold acclimation process. DEGs were enriched in pathways of photosynthesis, hormone signal transduction, and transcriptional regulation of plant-pathogen interactions. Further analyses indicated that decreased expression of *Lhca2* and higher expression of *SnRK2.8* are correlated with cold tolerance in SCZ.

**Conclusions:**

Compared with CSA, CSS was significantly more resistant to freezing after cold acclimation, and this increased resistance was associated with an earlier expression of cold-induced genes. Because the greater transcriptional differentiation during cold acclimation in SCZ may contribute to its greater cold tolerance, our studies identify specific genes involved in photoinhibition, ABA signal conduction, and plant immunity that should be studied for understanding the processes involved in cold tolerance. Marker-assisted breeding focused on the allelic variation at these loci provides an avenue for the possible generation of CSA cultivars that have CSS-level cold tolerance.

**Electronic supplementary material:**

The online version of this article (10.1186/s12864-019-5988-3) contains supplementary material, which is available to authorized users.

## Background

Low temperature, as an abiotic stress, restricts the growth, geographic distribution, and productivity of crops. Plant cold injury yields various symptoms, including stunted bud, wilting/yellowing of leaves, and tissue death [1]. Perennial plants have developed strategies to tolerate the cold stress in seasonal cycles. However, a sudden drop to freezing temperatures can cause severe damage or death even in cold-tolerant plants. Cold acclimation can help avoid this cold damage and has been the subject of experiments for many decades [2–5]. Cold acclimation is an induced adaptive process that increases freezing tolerance and is achieved by exposing the plant to various low temperatures above 0 °C for various periods of time, depending on the plant. Cold acclimation activates a systemic response to cold stress, and can be integrated with control of seed germination by the process called vernalization [6], which blocks premature germination during autumn warm spells. Hence, the study of cold acclimation is of wide interest in plant biology and agriculture.

Mechanisms of cold acclimation have been investigated especially well in cereal crops such as wheat and in model plants like Arabidopsis [3]. These studies identified biochemical changes associated with cold acclimation, including an increased generation of reactive oxygen species (ROS), an up-regulation of antioxidant enzymes, alteration in plant hormone levels, increases in metabolites such as γ-aminobutyric acid and soluble sugar, and accumulation of amino acids with osmotic effects, such as proline [3]. Cold acclimation and cold tolerance are associated with multiple biological processes and complicated regulatory networks [2, 7]. For instance, lipid membranes change in response to cold, including their glycoprotein content, lipid profile, membrane fluidity and structural organization [8–10]. Plant hormones, including abscisic acid (ABA) and gibberellin (GA), have long been known to be involved in cold response [11, 12]. Current cold responsive genes mostly originate from ancient cold responsive genes that have been conserved during the diversification of land plants, and have been especially vital in plant lineages that have undergone long-term radiation from tropical regions to temperate regions [13].

Investigations in many plant species have shown that numerous genes are up- or down-regulated during cold treatment, and these studies have been particularly informative in Arabidopsis [2, 11, 14, 15]. These studies show that gene regulation in cold acclimation and freezing stress acts at multiple levels. For instance, removal of repressive histone modifications on chromatin by HOS15, a WD40-repeat protein, has been shown to increase transcription of cold-regulated (COR) genes [16]. Epigenetic regulation of VERNALIZATION INSENSITIVE3 and FLOWERING LOCUS C is also involved, and requires both cold acclimation and the absence of daily warm temperature (15 °C or higher) [17]. C-repeat-binding factors 1–3 (CBF1–3) are transcription factors that integrate cold signals with altered gene expressions, including such COR genes as mitogen-activated protein kinases (MAPKs) [11, 18].

Compared with cereal crops and Arabidopsis, studies on cold acclimation in trees are relatively few. The tea plant (*Camellia sinensis* (L.) O. Kuntze) is widely planted to produce leaves for making tea. It is a perennial and evergreen tree that is restricted to temperate, subtropical and tropical production zones because tea plants are vulnerable to cold injury, especially during freezing winters. Low temperature appears to cause damage in the thylakoid membrane of cold-susceptible tea plants [19], as has been seen in other cold-susceptible species [20, 21]. The *C. sinensis* variety (var.) *sinensis* (CSS) and *C. sinensis* var. *assamica* (CSA) are two widely grown categories of germplasm, but CSS exhibits better cold resistance than CSA. Several publications have reported physiological responses, including gene expression, in response to cold stress in the tea plant [22, 23]. Early low temperatures (4–16 °C) may increase the cold resistance of tea plants to a later freezing winter [24]. A 4–5 °C acclimation has been reported to increase the activity of enzymes such as GR, SOD, and APX, and to the accumulation of high levels of sucrose, all proposed to be processes that could increase cold resistance [25, 26]. The contents of numerous metabolites in leaves were affected by low temperature, including products derived from sucrose hydrolysis that increase under cold treatment [27]. Changes in some small RNAs were associated with responses to low temperature (4 °C) after 1–48 h in the tea plant [28]. The transcriptome profiles in leaves of tea variety *Camellia sinensis* (L.) O. Kuntze cv. Longjing 43 were investigated under field conditions during overwintering [24]. All of these studies are limited to one or a few varieties under a few environments, so they lack any power to identify global responses that are conserved across environments and germplasm variation. Hence, our understanding of the intricacies of gene expression and regulation mechanisms underlying cold adaptation are still highly deficient for *C. sinensis*.

In this study, we comprehensively profiled dynamic transcriptomes via high through-put RNA-Seq analysis of tea leaves across controlled cold acclimation to investigate gene expression. The treatments were a 6 h short-term cold stimulus (CS), a 7-day long-term chilling acclimation (CA), a 7-day long-term freezing acclimation (FA), and a 7-day long-term de-acclimation (DA). To discover the gene expression responsible for higher cold resistance, we compared the transcriptomes in two tea varieties, CSS cv. Shuchazao (SCZ) with a high cold tolerance and CSA cv. Yinghong9 (YH9) with weak tolerance. Our results reveal the key regulated genes and their gene ontologies. We identify the key transcriptional pathways for the cold stimulus response, for chilling acclimation and for freezing acclimation in the tea plant. Interestingly, many of the genes identified are involved in photosynthesis, hormone signaling and plant immunity. Comparisons identified some key gene expression patterns associated with the capability for greater cold tolerance. Thus, our study provides novel insights into gene regulation from cold acclimation to cold adaptation in tea plants, and we discuss how this relates to similar investigations in other plant species.

## Results

### Different cold tolerance in two tea varieties during cold acclimation

**Fig. 1:**
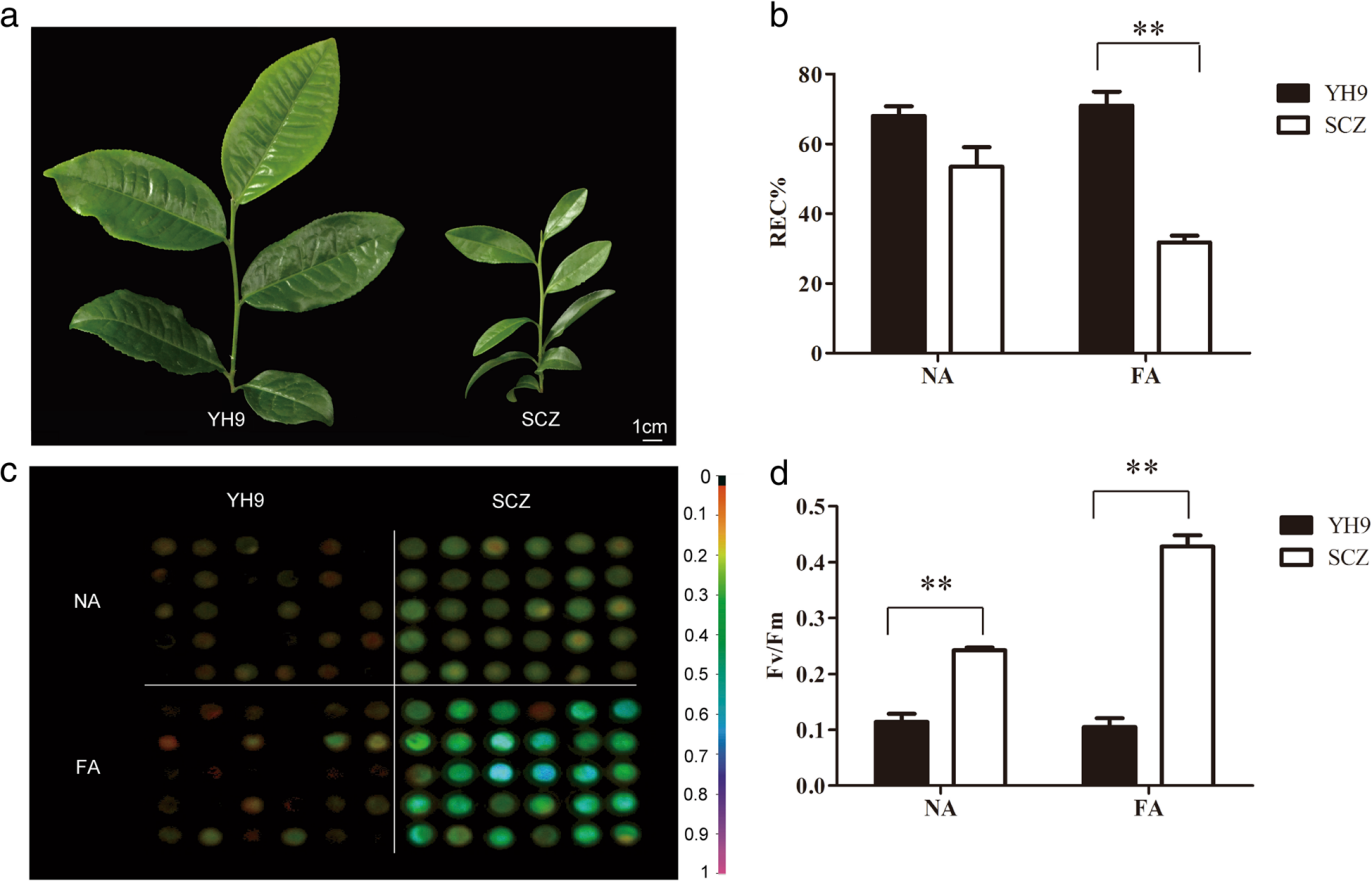
Different cold responses of two *Camellia sinensis* cultivars. The two tea varieties (**a**) were exposed to cold acclimation for two weeks followed by freezing stress. The relative electrical conductivity (REC) (**b**), chlorophyll fluorescence by Fv/Fm image (**c**), and Fv/Fm value (**d**) were examined. NA and FA represent no cold acclimation and treatment with freezing acclimation followed by − 5 °C for 12 h. Statistically significant differences between SCZ and YH9 were calculated using one-way ANOVA test using Statistical Package for the Social Sciences (SPSS) 16.0. ** *p* < 0.01

To investigate cold adaption in CSS and CSA, we assayed two cultivars with very different cold tolerances [26]. CSS tea variety SCZ is commonly planted in temperate and sub-tropical areas while cv. YH9, a CSA tea variety, is mainly planted in sub-tropical areas (Fig. 1a). Hence, SCZ is expected to have a higher cold tolerance than YH9, in agreement with the geographic difference in planting areas [26]. We treated both cultivars with − 5 °C freezing stress after FA treatment to confirm differences in cold tolerance. Relative electrical conductivity (REC), an indicator of cell leakage under freezing damage, was increased to more than 50% in both cultivars if there was no acclimation (NA), indicating that the freezing stress caused serious cell damage. However, REC was significantly lower in SCZ than YH9 if treated with freezing acclimation (FA), indicating a highly increased freezing tolerance in SCZ (Fig. 1b). The chlorophyll fluorescence (Fv/Fm) value was higher in SCZ than YH9 after treatment of FA (Fig. 1c, d). Together, this confirmed that cold acclimation can induce a higher freezing tolerance in cold resistant SCZ than in cold susceptible YH9.

### *C. sinensis* transcriptome profiles during cold acclimation

To characterize the transcriptomes in tea leaves in response to cold acclimation, we transferred one-year-old tea plants propagated from cuttings of each cultivar into a growth chamber for 4 weeks at 25 °C during the day and 20 °C at night to synchronize growth. We first treated the tea plants with CS for a short time (6 h), followed by a seven-day cyclic CA treatment at 10 °C during the day and 4 °C at night, and by subsequent seven-day FA treatment at 4 °C during the day and 0 °C at night. The tea plants were then grown at 25 /20 °C, the same as before the CS-CA-FA treatments, for 7 days to provide the DA treatment. The control group was the plants with NA, which were received no CS, CA, FA or DA treatments.

To understand gene expression during cold acclimation and de-acclimation, we examined the transcriptome profiles in tea leaves by using RNA-Seq analysis [24]. The mRNA of each sample was sequenced on an Illumina HiSeq X 10 platform. More than 41 million 150-bp paired-end reads were generated for each sample, totaling 479,445,976 and 466,081,620 clean reads from SCZ and YH9, respectively. We aligned the RNA-Seq reads to the *C. sinensis* draft genomic assembly [29] with tool HiSAT2 and StringTie to assemble and compare transcripts [30]. Approximately 81% and ~ 84% of reads were mapped onto the genome assembly for SCZ and YH9, respectively (Additional file 1: Table S1). A slightly higher mapping ratio was found in YH9, probably because YH9 is a *C. sinensis* var. *assamica,* as is the draft genome assembly utilized [29]. We obtained 85,949 and 87,842 transcripts, from 46,223 and 46,736 gene loci in SCZ and YH9, respectively (Additional file 1: Table S1). Of these candidate genes, 9272 from SCZ (20%) and 9785 from YH9 (21%) were not predicted as genes in the draft genome assembly [29]. The RNA-Seq data and transcript assemblies have been deposited and are publicly available in SRA and TSA at NCBI (https://www.ncbi.nlm.nih.gov/) under bio-project accession PRJNA387105.

### Overview of differential gene expression during cold acclimation

**Fig. 2:**
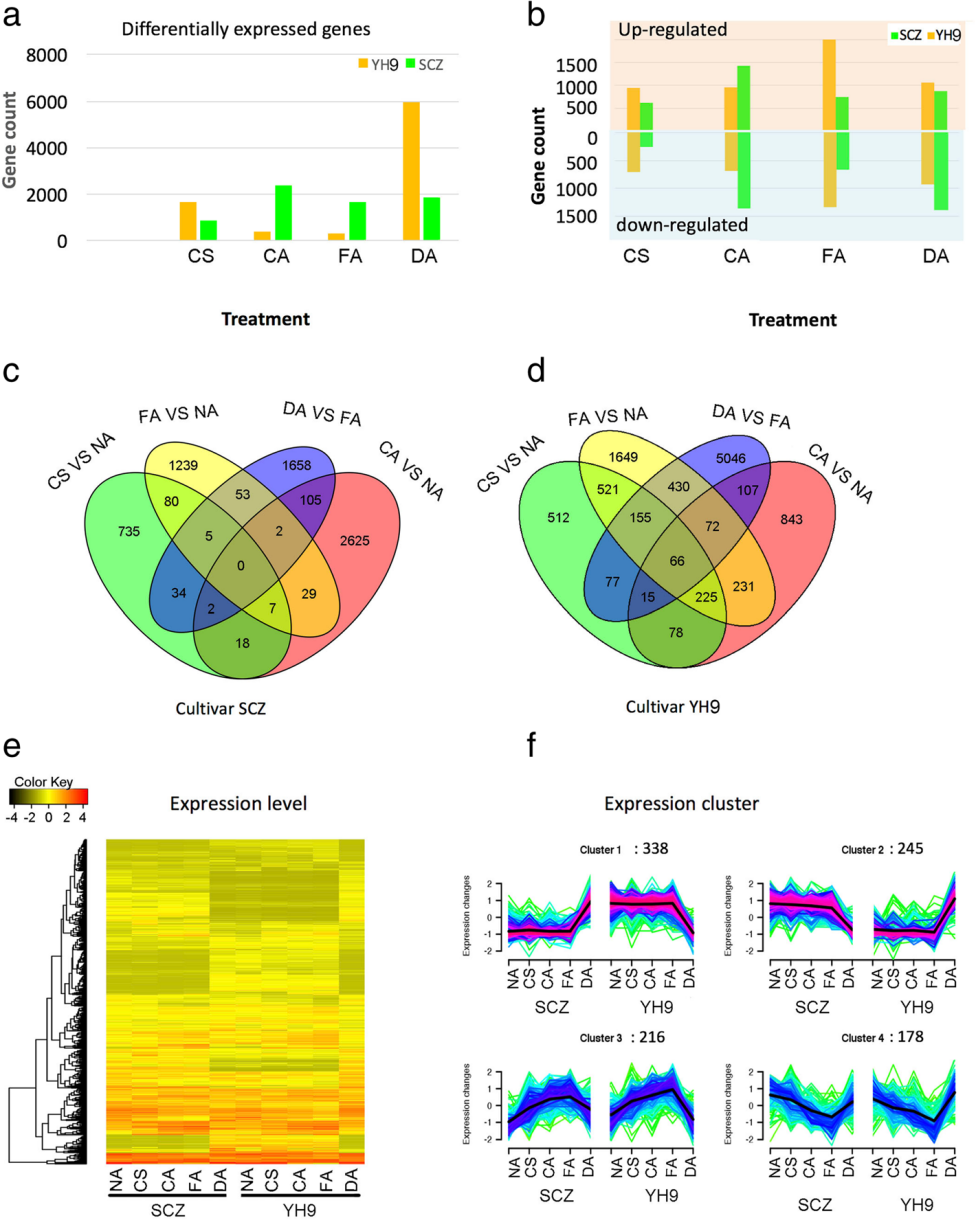
Differential expression of genes during chilling stimulus, chilling acclimation, freezing acclimation and de-acclimation. Image (**a**) shows the number of differentially expressed genes (DEGs) compared with the stage just before the corresponding treatment. Image (**b**) shows the number of DEGs in each stage compared with stage NA. Venn diagram images (**c** and **d**) show the number of DEGs across treatments. Image (**e**) shows the expression level of DEGs. Image f shows the clusters of expression patterns of DEGs. The change of expression level is shown after log10 conversion

To understand gene expression in response to cold acclimation, we measured transcript levels in terms of fragments per kilo-base exon per million reads (FPKM) based on RNA-Seq data. Genes were called Differentially Expressed Genes (DEGs) if they exhibited an at least 2-fold change in transcript abundance (Wald test, *p* < 0.05) using R package DESeq2 [31]. First, we investigated DEGs specific to each cold treatment by comparing with the stage immediately before the corresponding treatment. The CS group was treated with a short-term cold stimulus, from which 879 and 1589 DEGs were discovered in SCZ and YH9, respectively, compared with the control NA, indicating genes that are regulated for a rapid response to cold stimulus (Fig. 2a). The CA group was treated with 7d chilling acclimation, which generated a 2383 and 381 DEGs in SCZ and YH9, respectively (Fig. 2a), suggesting chilling acclimation genes. The FA group was treated with subsequent 7d freezing acclimation, which led to 1684 and 297 DEGs not observed in the CA group in SCZ and YH9 (Fig. 2a), respectively, suggesting responsive genes to freezing and cold resistance. The DA group, experiencing potential de-acclimation, yielded 1857 and 5968 DEGs that were not observed at the previous stage FA in SCZ and YH9, respectively (Fig. 2a). Fewer DEGs were found in the short-term and recovering treatment, while more DEGs in CA and FA treatment were observed in SCZ than YH9 (Fig. 2a).

In total, 6072 and 7749 DEGs were identified in cultivar SCZ and YH9 in response to one or more of the four treatments. Of these DEGs, 978 common DEGs were present in both cultivars (Fig. 2e). Their expression patterns fell into four major clusters (Fig. 2f). A total of 583 DEGs in cluster 1 (338 genes) and cluster 2 (245 genes) showed different patterns between the two tea cultivars in response to cold acclimation (Fig. 2f), indicating that these shared common DEGs responded differently and thus are candidate contributors to the difference in cold resistance between the two cultivars. The 394 genes in cluster 3 and 4 showed similar patterns in each cultivar (Fig. 2f).

We analyzed the DEGs after sequential CS, CA, and FA treatments using the starting stage NA as a control. Results revealed that the CA treatment led to the most DEGs in SCZ while the FA treatment led to the most DEGs in YH9 (Fig. 2b), suggesting an earlier peak time of most DEGs was associated with greater cold resistance in SCZ compared to YH9. Regarding the DEGs after the DA treatment, we used the FA treatment as the control, suggesting that these DEGs were responsible for recovering from cold acclimation. Comparison showed that 25 DEGs were shared by both CS and CA treatments while the number was reduced to 7 DEGs after further FA treatment in SCZ. Hence, these 25 common DEGs could be crucial for long-term CA or FA. The 7 common DEGs didn’t exhibit differential expression in the DA treatment, further suggesting these genes were responsible for cold response only (Fig. 2c). In YH9, we discovered 384 common DEGs after CS and CA treatments and this number was reduced to 291 DEGs after FA treatment. Of the 291 DEGs, 225 DEGs were cold responsive only and not present after DA recovery treatment (Fig. 2d).

### Functional comparison of cold responsive DEGs

To gain insights into the functions of discovered DEGs, we first assigned gene ontology (GO) term(s) to each DEG with the GO database (version 2016.04, http://geneontology.org) by similarity search using Blastx in the NCBI Blast+ package [32]. We then conducted an enrichment analysis using the statistical tool GOseq (version 1.18.0) [33]. DEGs relative to the NA stage were significantly (Wallenius test, *p* < 0.05) enriched for 317 and 700 GO terms in SCZ and YH9, respectively. The top 10 GO terms in each treatment, ranked by the number of DEGs, varied with treatment stages, suggesting altered functional responses at each stage.

**Fig. 3:**
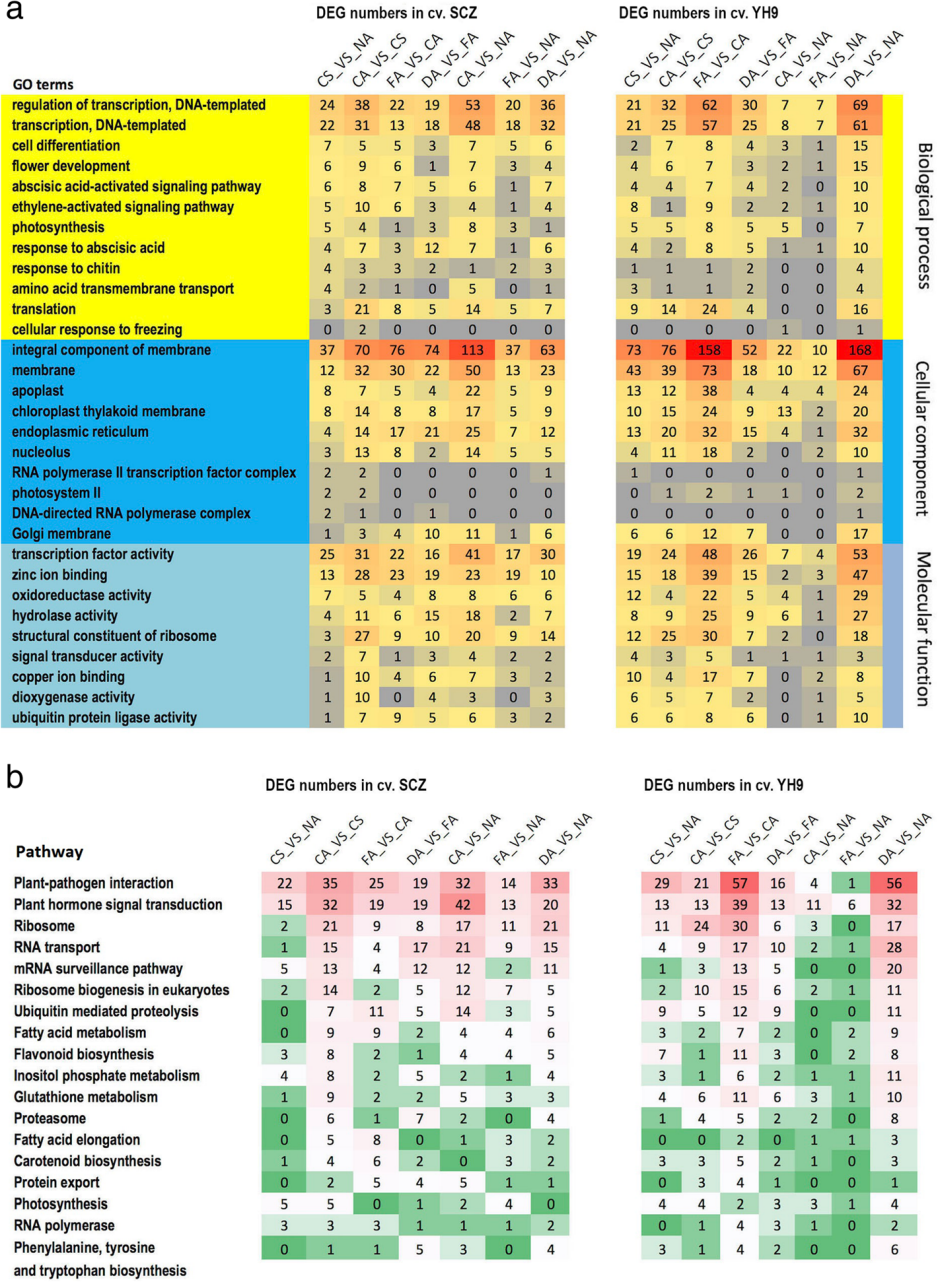
Comparison of top GO terms and pathways of DEGs in tea during cold stress, chilling acclimation, freezing acclimation and de-acclimation. The numbers of DEGs in each GO term are shown in three categories defined in the GO database (**a**). The numbers of DEGs in each pathway are shown for each treatment comparison (**b**). The comparison is described as A_vs_B, which means the DEG was found in the A treatment relative to the B treatment. NA, CS, CA, FA and DA indicate the no acclimation control, cold stimulus, chilling acclimation, freezing acclimation, and de-acclimation, respectively

GO terms of shared DEGs between the two cultivars could reflect common responses to cold acclimation. Thus, we assigned GO terminology to the shared 978 DEGs, and found that they belonged to 317 known GO terms. Of these GO terms, more were classified into the “biological process” category than the “cellular component” or the “molecular function” category. The top 10 enriched (Wallenius test, *p* < 0.05) GOs of common DEGs exhibited a very similar rank, although the DEG numbers in each GO term differed between cultivars (Fig. 3a). The shared top GO terms included “regulation of transcription”, “flower development”, “abscisic acid-activated signaling pathway”, and “photosynthesis” in the biological process category. The top enriched GOs in the cellular component category included “membrane”, “apoplast”, and “chloroplast thylakoid membrane”. The top enriched GOs in the molecular function category included “transcription factor activity”, “hydrolase activity”, “zinc ion binding” and “oxidoreductase activity” (Fig. 3a). We found that other GOs, besides the top enriched terms, also contributed to the responses to cold treatment. For instance, we found that GOs of two DEGs from the comparison CA_vs_ CS in SCZ were defined as cellular response to freezing (GO:0071497). GO terms for a DEG from the comparison CA_vs_NA in YH9 were also assigned to cellular response to freezing. The differences in “cellular response to freezing” may also reflect the difference in cold tolerances between cultivars.

### Enriched pathways associated with DEGs in response to cold acclimation

To understand the metabolic or signal pathways involved in cold treatment responses, we first mapped all DEGs to the Kyoto Encyclopedia of Genes and Genomes (KEGG) database to retrieve involved pathways in each cultivar. In total, 22 and 44 significantly (*p* < 0.05) enriched pathways were identified for all DEGs in SCZ and YH9, respectively. Then, we searched for the pathways shared by both tea cultivars and found 18 common enriched pathways (Fig. 3b), of which “Plant-pathogen interaction” had the most abundant DEGs enriched, while “Plant hormone signal transduction” and “Ribosome” were also ranked high. We found different numbers of DEGs in the same pathway in the two cultivars (Fig. 3b). Among the top five pathways, the most abundant DEGs appeared in the comparison CA_vs_CS, which was after the CA treatment, in SCZ while they appeared at a later stage in the comparison FA_vs_CA after the FA treatment in YH9, suggesting earlier altered gene expression in the shared pathways in highly cold-tolerant SCZ.

We also compared the pathways associated with DEGs relative to the starting stage NA. DEGs from the short-term CS treatment were mapped into 66 and 108 KEGG pathways in SCZ and YH9, respectively, but only eight pathways were significantly (*p* < 0.05) enriched in SCZ. We found the enriched pathways were different from the shared pathways mentioned above (Fig. 3b). The top three pathways ranked by the number of DEGs were “plant pathogen interaction (22 DEGs)”, “plant hormone signal transduction (15 DEGs)” and “photosynthesis (5 DEGs)” in SCZ. However, 13 significantly (*p* < 0.05) enriched pathways were found in YH9, and the top three pathways with the most DEGs were “Carbon metabolism (24 DEGs)”, “Glycerolipid metabolism (12 DEGs)” and “Phagosome (12 DEGs)”. This suggested fewer and more pathways were affected in the high and low cold-tolerant cultivar after the short-term CS, respectively. Moreover, the cold-sensitive cultivar appeared to be mostly changing expression of general metabolism genes, while the cold-tolerant cultivar was modifying general resistance/stress pathways.

After the long-term CA treatment, the top five of nine enriched (*p* < 0.05) pathways were “Plant hormone signal transduction” (42 DEGs), “Pyruvate metabolism” (18 DEGs), “Phagosome” (18 DEGs), “Glycolysis / Gluconeogenesis” (16 DEGs) and “Carbon fixation in photosynthetic organisms” (16 DEGs) in SC. In contrast, in YH9, the top five of nine enriched (*p* < 0.05) pathways were “Ribosome” (11 DEGs), “Carbon metabolism” (21 DEGs), “Phagosome” (17 DEGs), “Glyoxylate and dicarboxylate metabolism” (13 DEGs) and “Ribosome biogenesis in eukaryotes” (10 DEGs). After the subsequent long-term FA treatment, only two pathways termed “RNA degradation” (11 DEGs) and “Photosynthesis” (4 DEGs) were enriched in SCZ; however, 12 enriched pathways were found in YH9, including the top five pathways termed as “Carbon metabolism” (47 DEGs), “Phagosome” (24 DEGs), “Purine metabolism” (23 DEGs), “Pyruvate metabolism” (21 DEGs), and “Carbon fixation in photosynthetic organisms” (20 DEGs). This suggested that the final effects after chilling acclimation and freezing acclimation induced less change in pathways in SCZ, which matches the observed higher adaptation to cold and stable physiological responses in SCZ (Fig. 1).

### Key DEGs in the photosynthesis pathway

**Fig. 4:**
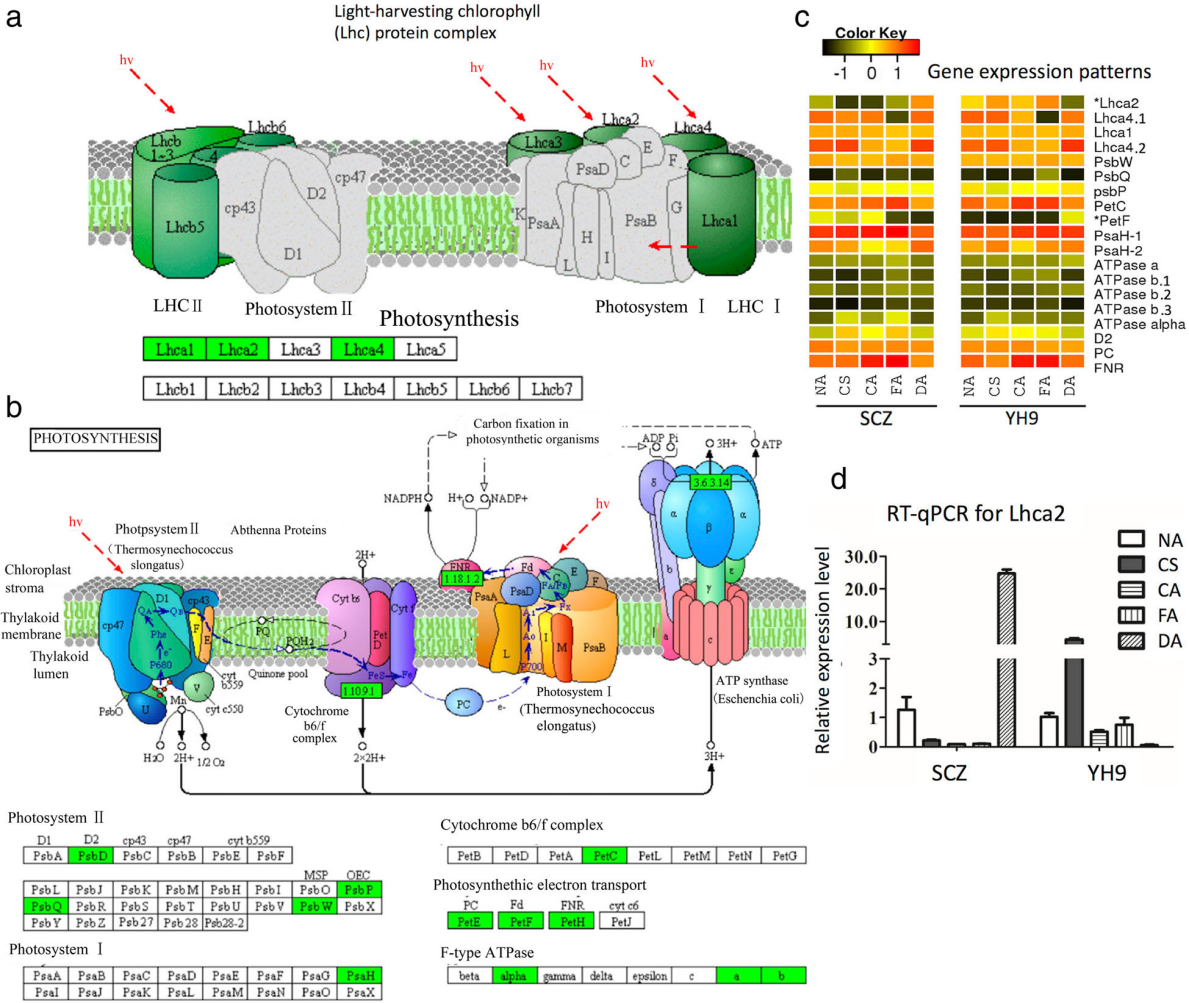
Differentially expressed genes involved in photosynthesis and chloroplast. Images (**a** and **b**) show differentially expressed genes shaded in a green box in the photosynthesis pathways depicted in KEGG (map 00195). Image (**c**) shows a comparison of gene expression patterns in SCZ and YH9. The heatmap was generated from the log2FPKM mean value calculated from two replicates of RNA-Seq data. The asterisk before a gene product name indicates the most different patterns between two cultivars. Image d shows the relative expression level of gene *Lhca2 validated from RT-qPCR*

As the photosynthesis pathway was enriched in DEGs, we further characterized functional annotation of these DEGs. The proteins encoded by the DEGs were mainly located in the chloroplast, and annotated to function as antenna proteins (Fig. 4a), photosystems I and II components, cytochromes, and electron transport (KEGG map 00062, Fig. 4b, Additional file 2: Table S2). Comparison of these DEGs’ expression between the two cultivars revealed 17 genes that had similar patterns of expression. In contrast, light-harvesting chlorophyll gene 2 (*Lhca2*) had quite different expression patterns in the two cultivars (Fig. 4c). *Lhca2* encodes a protein in the membrane complex of photosynthesis system I (Fig. 4a). *Lhca2* was highly expressed in YH9 but much less expressed in SCZ in response to cold treatments. During recovery from cold to warm temperature, *Lhca2* expression showed a reversed pattern, increasing in SCZ and decreasing in YH9 (Fig. 4c and d). We validated the *Lhca2* expression level using RT-qPCR (Fig. 4d, more validation in Additional file 3: Figure S1). Hence, low expression of *Lhca2* was negatively correlated with cold-tolerant capability.

### Regulation of key DEGs in plant-pathogen related immunity

**Fig. 5:**
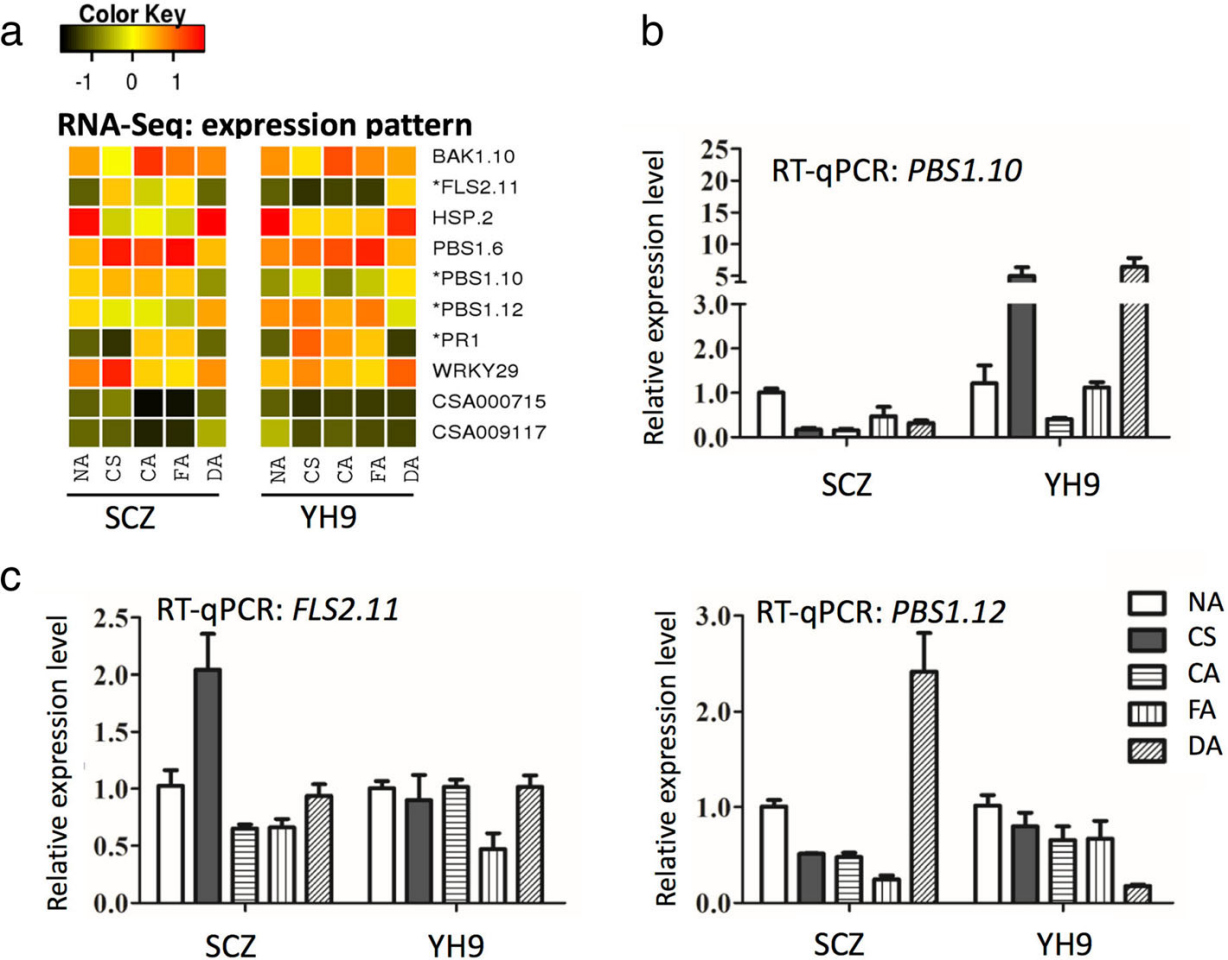
Expression patterns of DEGs associated with biotic defense in the tea plant. The heatmap (**a**) represents the gene expression patterns generated from the log_2_FPKM of the mean value of replicates from RNA-Seq data. The asterisk before a gene product name listed in the right side of each row indicates the most different patterns in RNA-Seq data from the two cultivars. The relative expression level of gene PBS1.10, PBS1.12 (**b**) and gene FLS2.11 (**c**). NA, CS, CA, FA and DA represent the cold treatments

We further characterized the identified DEGs involved in plant-pathogen related immunity in the KEGG database to locate the proposed regulatory pathway. We also used the UniProt database for additional functional annotation based on protein similarity. Ten common DEGs in this category were found in both SCZ and YH9 (Table 1); however, 70 and 56 DEGs were present only in SCZ and YH9, respectively, indicating different regulation in pathways in the two tea varieties. Many of the predicted functions of these DEGs are defense-related kinases, transcription factors, or heat shock proteins (Table 1). Multiple members of gene families *BAK1*, *HSP*, and *PBS1* were expressed, but only a subset of members was differentially expressed (Table 1), indicating functional diversity of these families in cold response. Among these genes, *FLS2* exhibited very different expression patterns between SCZ and YH9 (Fig. 5a, c).

**Table 1:** DEGs related to plant immunity shared between SCZ and YH9

Gene name	Pathway entry	Manually annotated function
CSA015695	K13416	brassinosteroid insensitive 1-associated receptor kinase 1 (BAK1.10)
CSA001565	K13420	LRR receptor-like serine/threonine-protein kinase (FLS2)
CSA030713	K04079	heat shock protein 83-like (HSP.2)
CSA017980	K13430	serine/threonine-protein kinase PBS1 (PBS1.6)
CSA007045	K13430	serine/threonine-protein kinase PBS1 (PBS1.10)
CSA024279	K13430	serine/threonine-protein kinase PBS1 (PBS1.12)
CSA025610	K13449	basic form of pathogenesis-related protein 1 (PR1)
CSA004479	K13426	WRKY transcription factor 29 (WRKY29)
CSA000715	NA	unknown
CSA009117	NA	unknown

The column “Gene name” represents the gene ID in the publicly available draft tea genome assembly. Pathway entry represents the ID in the database KEGG.

### Regulation of key DEGs in plant hormone signal transduction

**Fig. 6:**
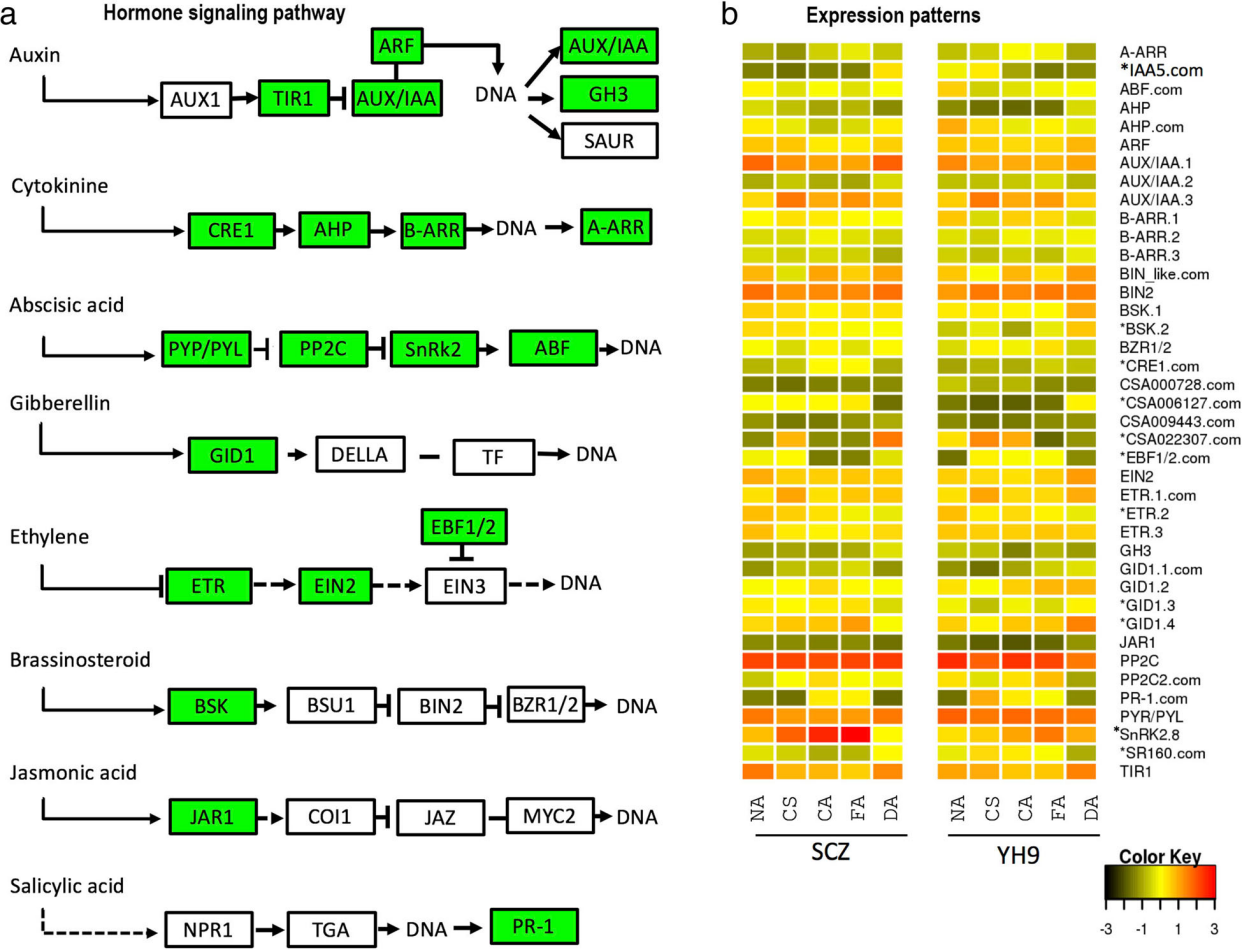
DEGs in hormone signaling pathways in response to cold treatment. **a** Green boxes show the mapped DEGs in plant hormone signaling pathways, as modified from a KEGG map (ID 04075). **b** The expression patterns are shown as a heatmap. The most different patterns of expression between the two tea cultivars are marked by asterisks. The shared DEGs in the cultivars are tagged by the suffix .com. NA, CS, CA, FA and DA denote the cold treatments

We identified 115 DEGs involved in hormone signal transduction from both cultivars of SCZ and YH9 in response to cold treatments. Of those, 15 DEGs were shared in the two tea cultivars, while 59 and 41 DEGs were only present in the respective SCZ and YH9, indicating both common and cultivar-specific regulation under cold treatments (Table 2). The DEGs were mapped into hormone signal transduction pathways of auxin, cytokinin, abscisic acid, gibberellin, ethylene, brassinosteroid, jasmonic acid and salicylic acid in *Arabidopsis* and *Populus* (Table 2, Fig. 6). Most of the known genes in auxin, cytokinin, abscisic acid and ethylene signal pathways were identified as DEGs in this study (Fig. 6a). Different expression patterns of common DEGs were found between the two tea cultivars in response to cold treatments (Fig. 6b), indicating regulation of these common DEGs was also associated with different cold tolerances. The expression of *SnRK2.8*, functioning in ABA signal transduction, osmotic stress and glycol-metabolism [34], was significantly higher in SCZ than YH9 in response to cold acclimation (Fig. 6b).

**Table 2:** DEGs involved in plant hormone signal transduction

Gene name	KEGG #	Manually annotated function	Shared DEG
CSA026554	K14492	two-component response regulator ARR-A family (A-ARR)	
CSA025614	K21568	auxin-responsive protein IAA5-like (IAA5)	Yes
CSA003423	K14432	ABA responsive element binding factor (ABF)	Yes
CSA025810	K14490	histidine-containing phosphotransfer protein (AHP)	
CSA014553	K14490	histidine-containing phosphotransfer protein (AHP)	Yes
CSA002625	K14486	auxin response factor (ARF)	
CSA014562	K14484	auxin-responsive protein IAA (AUX/IAA.1)	
CSA019710	K14484	auxin-responsive protein IAA (AUX/IAA.2)	
CSA023080	K14484	auxin-responsive protein IAA (AUX/IAA.3)	
CSA027576	K14491	two-component response regulator ARR-B family (B-ARR.1)	
CSA036208	K14491	two-component response regulator ARR-B family (B-ARR.2)	
CSA036403	K14491	two-component response regulator ARR-B family (B-ARR.3)	
CSA015695	K14502	brassinosteroid insensitive 1-associated receptor kinase 1-like (BIN_like)	Yes
CSA001186	K14502	protein brassinosteroid insensitive 2 (BIN2)	
CSA007850	K14500	BR-signaling kinase (BSK.1)	
CSA032457	K14500	BR-signaling kinase (BSK.2)	
CSA022935	K14503	brassinosteroid resistant 1/2 (BZR1/2)	
CSA016248	K14489	cytokinin receptor (CRE1)	Yes
CSA000728	NA	Unknown	Yes
CSA006127	NA	hypothetical protein	Yes
CSA009443	NA	Unknown	Yes
CSA022307	NA	Unknown	Yes
CSA029041	K14515	EIN3-binding F-box protein (EBF1/2)	Yes
CSA008678	K14513	mitogen-activated protein kinase kinase 4/5 (EIN2)	
CSA031389	K14509	ethylene receptor (ETR.1)	Yes
CSA024687	K14509	ethylene receptor (ETR.2)	
CSA004165	K14509	ethylene receptor (ETR.3)	
CSA033915	K14487	auxin responsive GH3 gene family (GH3)	
CSA012511	K14493	gibberellin receptor GID1 (GID1.1)	Yes
CSA020494	K14493	gibberellin receptor GID1 (GID1.2)	
CSA020665	K14493	gibberellin receptor GID1 (GID1.3)	
CSA021414	K14493	gibberellin receptor GID1 (GID1.4)	
CSA009988	K14506	jasmonic acid-amino synthetase (JAR1)	
CSA010349	K14497	protein phosphatase 2C (PP2C)	
CSA028964	K14497	Highly ABA-induced PP2C gene 2 (PP2C2)	Yes
CSA025610	K13449	pathogenesis-related protein 1 (PR-1)	Yes
CSA027144	K14496	abscisic acid receptor PYR/PYL family (PYR/PYL)	
CSA006311	K14498	serine/threonine-protein kinase SRK2 (SnRK2.8)	
CSA007707	NA	systemin receptor SR160	Yes
CSA036164	K14485	transport inhibitor response 1 (TIR1)	
CSA022307	K13415	protein brassinosteroid insensitive 1 (BRI1)

The k number in the KEGG column is the pathway entry in the KEGG database. The abbreviation of the gene in parenthesis was added with a dot and digital suffix if multiple copies of DEGs were annotated as the same function.

## Discussion

Identifying the genes responsible for high cold resistance could help the breeding of new cultivar to extend tea plantations into cooler zones. To date, the expression of several genes [26] or miRNAs [28] were reported in response to cold stress in tea plant, but these studies did not take acclimation or cultivar differences into account. The transcriptome profile under natural temperature changes was reported in a tea variety between winter and early spring [24]. Here, the systematic transcriptome profiling in tea varieties responsible for high or low cold resistance capability under controlled cold acclimation was investigated.

From the DEG comparisons between SCZ, with a high cold resistance, and YH9, with a low cold resistance, we conclude that at least two expression features associate with higher cold tolerance. First, less expression fluctuation in a short-term CS may reflect higher tolerance. This could be seen from less DEGs (879 vs. 1589) in SCZ than in YH9 after short-term (6 h) CS. Second, more DEGs could be induced by a long-term cold acclimation in the greater cold tolerant variety, which may result in a better preparation for freezing stress, and thus a higher cold tolerance. This is demonstrated by 2383 and 381 DEGs in SCZ and YH9, respectively, after 7-day long-term cold acclimation. The response of DEGs during the long-term cold acclimation, not the short-term CS, appears to be crucial for acquiring higher cold resistance. Future studies on the function of these 25 DEGs in SCZ during the long-term cold acclimation should be of highest priority, including analysis by overexpression and/or knock-out mutations. However, the transformation of tea plants is still a big challenge.

Cold acclimation is a complicated biological process that is expected to integrate multiple signals. Here, we identified DEGs involved in many pathways, partly confirming that complexity. Signal transduction pathway is known to play crucial roles in response to the stress of low temperatures [35]. Evidences have established that Ca^2+^ influx was a very important change in membrane during cold stress [36]. Some genes have been shown to sense changes in intracellular Ca^2+^ levels via phosphorylation to initiate downstream signaling processes [37]. In our study, we found related gene regulation for Ca^2+^ signaling. Calmodulin (*CaM*) gene was specifically found in SCZ and up-regulated during cold stress. Two calcineurin B-like protein (*CBL*) genes, three calcium-dependent protein kinase (*CDPK*) genes, one calmodulin-like protein (*CML*) genes and three CBL-interacting protein kinase (*CIPK*) genes were up-regulated during cold treatment in SCZ while only two *CBP* genes were up-regulated in YH9 (Additional file 4: Table S3). In addition, fifteen receptor like kinase genes for phosphorylation were induced during cold treatment in SCZ and six were induced in YH9. Therefore, Ca^2+^ signaling-related genes might contribute to higher cold tolerance in SCZ than YH9.

*FLS2*, encoding a type of LRR receptor-like protein kinase, which exhibited a higher expression level in only SCZ, was a DEG involved in plant-pathogen related immunity in the KEGG database. It is known that abiotic stresses induce plant immunity gene expression [38], perhaps as a way to minimize biotic infestation after abiotic tissue damage. We speculate that cold-induced plant immunity may result from a common signaling process like those for other abiotic stresses. This signaling could include components in plant hormone cross-talk. A LRR receptor-like protein kinase (*GsLRPK*) in *Glycine soja* is a positive regulator to cold stress tolerance. Overexpression of *GsLRPK* in Arabidopsis can increase the expression of many COR gene markers [39].

MAPK cascades are involved in plant signal transduction in response to cold [40], which could relay and amplify signals via three types of reversibly phosphorylated kinases leading to the phosphorylation of substrate proteins [41]. Here, we identified one MAPK gene was up-regulated in SCZ while down-regulated in YH9 (Additional file 4: Table S3).

We found that the enriched GO terms for DEGs included the “abscisic acid-activated signaling pathway”. ABA signaling pathway has been identified as a central regulator of abiotic stress response in plants, triggering major changes in gene expression and adaptive physiological responses [42]. Zeaxanthin epoxidase (*ZEP*), 9-cis-epoxycarotenoid dioxygenase (*NCED*) and abscisic aldehyde oxidase (*AAO*) are three key enzymes involving in ABA synthesis [43]. In our study, there are two *ZEP* genes were up-regulated during cold treatment in SCZ while down-regulated or constant in YH9. *NCDE* 1 gene was up-regulated but not significant in SCZ. *SnRKs* allowed intensifying activation of the ABA-induced signaling pathway through a double negative regulatory system (*PYR/PYL-| PP2C-| SnRK2*) [44]. Here, one *SnRK2.8* gene was dramatically up-regulated during CS and CA in SCZ (Additional file 4: Table S3). Expression of most key genes in plant hormone signaling pathways were significantly altered in expression (Fig. 6), suggesting a huge change in hormone signaling. Plant hormone pathways are known to cross-talk under stress [45]. Hence, the CA and FA results observed agree with hormonal cross-talk manner that can increase the cold tolerance and thereby minimize cold damage.

Transcription factors (TFs) play important functions in plant development and stress tolerance. Our transcriptomic data depicted some TFs that govern the massive and highly coordinated transcriptional changes under short/long term cold stress in SCZ and YH9 (Additional file 5: Table S4). Consistent with findings in other plant exposed to cold stress, some of the major TFs of SCZ and YH9 were *AP2*, *ERF*, *bHLH*, *bZIP*, *MYB*, *NAC*, *WRKY*, *MADS*, *CAMTA*, *B3-ARF*, *ZAT*, *C2C2-Dof*, indicating the complexity of the regulatory pathways during cold acclimation [24, 46]. Transcripts encoding TFs like *ERF* (CSA013480), *NAC* (*NAC*86, 94), *WRKY* (*WRKY*42, 50), *MYB*86, *bHLH* (*bHLH*36, 48, 102, 116, 118), *MADS- M-type*, *B3-ARF*, and *ZAT* were significantly upregulated into a higher level in tolerant SCZ than sensitive YH9 when expose to cold stress. These TFs are reported to be linked to cold stress resistance in plants possibly via activating ABA-independent and ABA-dependent pathway in plant [47–50]. The highly up-regulated expression of TFs in SCZ could play a vital role in higher cold tolerance in SCZ.

CBF/dehydration-responsive element-binding factor (*DREB1*) from *AP2/ERF* super-family (*CBF1/DREB1B*, *CBF2/DREB1C*, and *CBF3/DREB1A*) in Arabidopsis plays a prominent role in cold acclimation [7, 51]. *CBF* expression was positively correlated with cold tolerance, and expression of downstream CORs can be activated by binding with DRE/CRT cis-acting elements [52]. Here, we found consistent results in tea plant. Expression of three *CBF* genes were found to be significantly induced during cold acclimation. The *CBFs* expression level in SCZ was significantly higher than that in YH9, the *CBF* pathway may play an important role in tea plants**.** Many *COR* genes were induced during the cold acclimation in both cultivars (Additional file 6: Table S5). Most *COR* genes have similar expression levels, which indicates that these *COR* genes are conservative in regulating of defense responses in tea plant. COR genes encoding *LEA2 (*MSTRG.46934*), HSP70* (CSA030367), *PRP* (CSA014200, CSA008621), *CIPs, PEIs, TLPs* (CSA000129, CSA001426), *ChiA* (CSA012225) and endoglucanase (endoglucanase 6, 8, 11, 14, 17) had higher transcript abundance in SCZ than YH9. Therefore, both CBFs and these CORs may contribute to the higher cold tolerance after cold acclimation in SCZ.

ROS is a toxic compound that can cause oxidative damage to proteins, DNA, lipids and other cellular components [53, 54]. ROS-scavenging enzymes, such as *SOD*, *POD, APX*, monodehydroascorbate reductase (*MDAR*), glutathione peroxidase (*GPX*) and glutathione S-transferase (*GST*), were found in our transcriptome’s data, but only *POD* (CSA023255, CSA026001) and *GST* (CSA006422) were up-regulated in SCZ. This indicates that SCZ suffers less cold damage than YH9 during cold acclimation, consistent with observed higher cold tolerance in SCZ. Flavonoids has ROS-scavenging activity that protects against oxidative damage and controls ROS levels under abiotic stresses [55]. This was supported by increased flavonoids in winter sweet spinach [56] and transcription of genes for biosynthesis of flavonoids correlated with freezing tolerance after cold acclimation in in *Arabidopsis* [57]. In this study, the flavoid associated genes involved in flavonoid biosynthesis were detected between SCZ and YH9 (Additional file 7: Table S6). During CA treatment, flavonol synthase (*FLS)* was significantly up-regulated only in SCZ with high fold change, while anthocyanidin synthase (*ANS*) was up-regulated in YH9. Shen et al. also found that *FLS* expression was up-regulated under low temperature stress in SCZ [58]. Thus, both *FLS* and *ANS* could contribute to enhance cold tolerance in tea plant but with difference. The increased fatty acid desaturation in membrane lipids allows functional membrane fluidity to be maintained at low temperature [19, 59]. *SAD*, *FAD* and *LTP8* are responsible for fatty acids unsaturation. In this study, *SAD* and *LTP8* were both significantly up-regulated in SCZ during CA and FA treatment. Interestingly, the expression of *FADs* had different pattern in two cultivars. We speculate that the higher *SAD* and *LTP8* expression were contribute to higher cold resistant in SCZ (Additional file 8: Table S7).

To maintain osmotic equilibrium, plants accumulate a series of compatible solutes, including soluble sugars and low molecular weight compounds such as proline and glycine betaine during cold stress response [2, 60, 61]. In our previous studies, we found SCZ exhibited a higher accumulation level of soluble sugars, particularly sucrose than YH9 during cold acclimation [26]. In this study, inductions of sucrose biosynthesis-related genes (*SUS*, *SPS*) and most of *TPS* were considerably higher in SCZ than YH9. The enzymes β-amylase (*BMY*) degrades starch to soluble sugar which leads to increased maltose, glucose, fructose and sucrose levels after further conversion. Cold stress also induces *BMY* transcript level or activity [62]. Here, *BMY* was significantly up-regulated in both cultivars, but the expression level in SCZ was higher than that in YH9. *HXK* is known to phosphorylate glucose and fructose, and participate in sugar signal transduction by modulating the abundances of diverse gene transcripts and integrating stress response substrates, including ABA and ethylene [63, 64]. *HXK* was induced in *Jatropha curcas* when exposed to cold stress [65]. Similarly, *HXK* was induced more in SCZ than YH9. To date, it is known that the proline biosynthesis is catalyzed by *P5CS* and *P5CR* in plants [66]. In this study, expression level of *P5CS* and *P5CR* were higher in SCZ than YH9 (Additional file 9: Table S8). Therefore, these genes are also involved in cold acclimation for conveying better tolerance.

Photoinhibition is a decline in photochemical activity when the available light exceeds the real receptive capacity of the photosynthesis. Exposure to low temperature has been found to cause photoinhibitory stress in chilling-sensitive plants [67]. In spinach, photoinhibition has been seen to lead to the accumulation of osmoprotectant polyamines by increasing S-adenosylmethionine decarboxylase activity [68]. In maize, light is necessary for effective cold acclimation under low temperature [69], suggesting an involvement of photoinhibition. Here, we identified many acclimation-induced DEGs enriched in photosynthesis, a novel discovery for the tea plant. The expression of light capture complex genes was decreased, suggesting a decline in photochemical activity. The expression of light-harvesting chlorophyll *Lhca2* in photosystem I (Fig. 4) may be crucial for cold tolerance. Under cold treatment, the decreased expression of *Lhca2* in cold-resistant SCZ contrasted with the increased expression in cold-susceptible YH9 suggesting that *Lhca2* expression could be a good indicator of cold resistance. Here, we conclude that low expression of *Lhca2* suggests photoinhibition and indicates a higher cold tolerance in tea plants. This is further supported by a recent finding that photoinhibition is necessary for acquiring cold resistance in maize, a chilling-sensitive plant [69]. The reduced expression of *Lhca2* may lead to low activity of LHCA2 protein in photosynthesis system I, suggesting that the absorption of light is reduced and that light suppression at low temperatures is alleviated, thereby improving cold resistance.

## Conclusion

This study provides a comparative transcriptomic analysis of how gene expression differs in cold acclimated leaves of two tea varieties with different tolerances to cold stress.

**Fig. 7:**
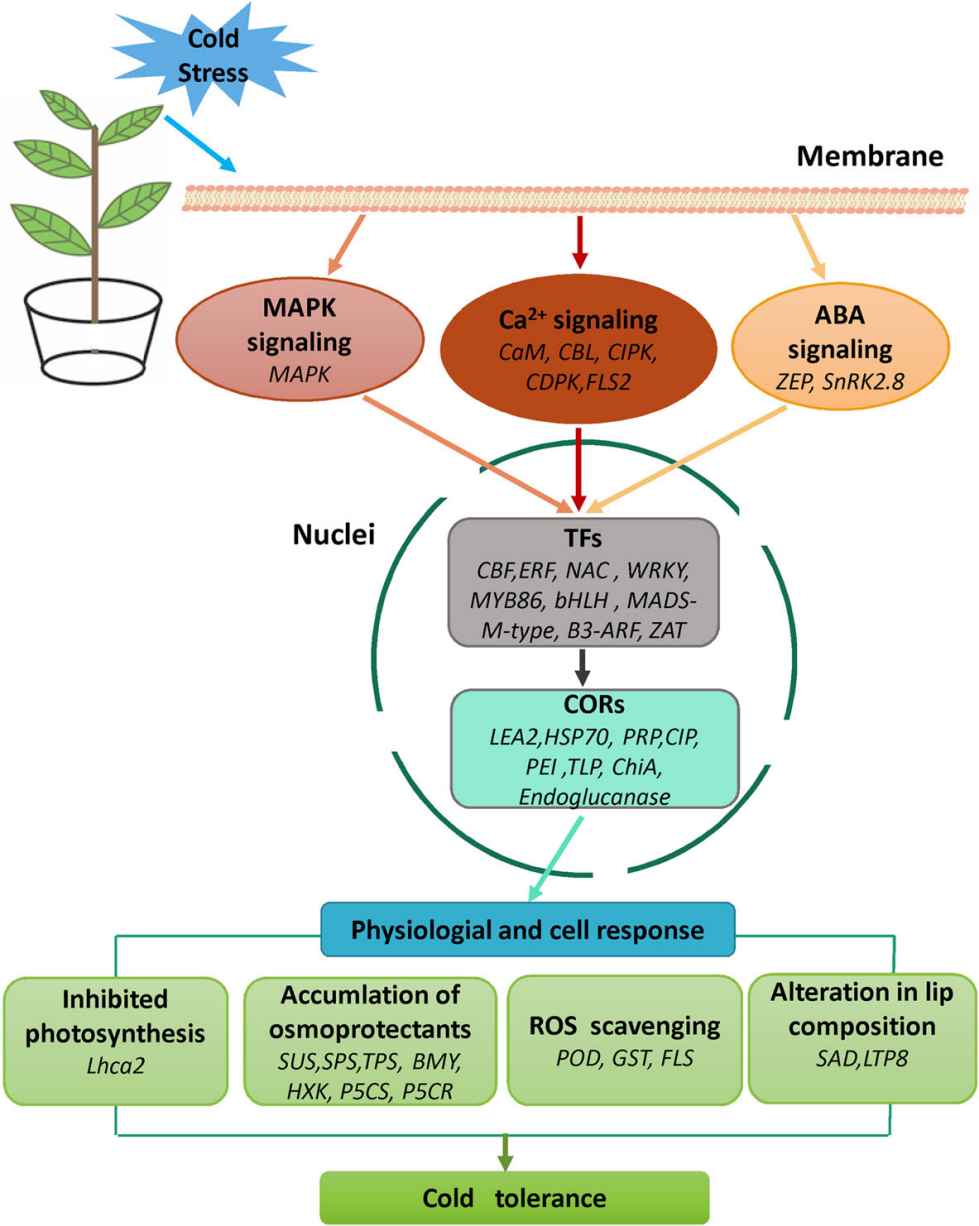
A schematic signaling and involved genes for achieving cold tolerance in tea plant (SCZ)*.* MAPK, mitogen-activated protein kinase; CaM, calmodulin; CBL, calcineurin B-like Ca^2+^ sensors; CIPK, CBL-interacting protein kinase-calcineurin B-like protein; CDPK, Ca^2+^ − dependent protein kinase; FLS, LRR receptor-like serine/threonine-protein kinase; ZEP, zeaxanthin epoxidase; SnRK2.8, SNF1-related protein kinase 2.8; CBF, C-repeat binding factors; ERF, ethylene response factor; NAC, NAC domain transcription factor; WRKY, WRKY transcription factor; MYB86, MYB transcription factor86; bHLH, basic helix-loop-helix protein; MADS-M-type, MADS-box domain protein; B3-ARF, B3 domain transcription factor; ARF, auxin response factor; ZAT, zinc finger protein; LEA2, late embryogenesis abundant protein 2; HSP70, heat shock protein 70; PRP, proline-rich protein; CIP, cold-induced protein; PEI, pectinesterase inhibitors; TLP, thaumatin-like protein; ChiA, chitinase; SUS, sucrose synthase; SPS, sucrose phosphate synthase; TPS, trehalose phosphate synthase; BMY, beta-amylase; HXK, hexokinase; P5CS, proline-5-carboxylate synthetase; P5CR, proline-5-carboxylate reductase; POD, peroxidase; GST, glutathione S-transferase; SAD, stearoy-l ACP desaturase; LTP8, lipid-transfer protein 8

A responsive molecular model of cold stress in the *CSS* was summarized in Fig. 7. The cold signal is perceived and then mainly transduced further by Ca^2+^ signaling and ABA signaling resulting in the activation of downstream TFs. The activation of *COR* genes through involvement of TFs leads to trigger a series of physiological and cell response, and improved tolerance to cold stress. The greater transcriptional differentiation during cold acclimation in SCZ may explain its better cold tolerance compared to YH9. Genes involved in photoinhibition, hormone signaling, and plant immunit*y* are now identified as logical targets for further investigation and for future development of improved cold-tolerance in tea cultivars.

## Methods

### Plant materials and treatments

Cuttings of *C. sinensis* var. *sinensis* cv. Shuchazao and *Camellia sinensis var. assamica* cv*.* Yinghong9 were obtained from the Dechang Tea Plantation in Anhui (116° 56′ 24″ E, 31° 27′ N) and the Tea Research Institute of Guangdong Academy of Agricultural Sciences (113° 22′ 48″ E, 24° 10′ 12″ N), China, respectively. One-year-old clonal plants propagated from cuttings were transferred to a growth chamber with temperature cycles of 25 °C in the day and 20 °C at night, 12 h photoperiod, and 70% relative humidity for 1 month. Subsequently, they were subjected to treatments for cold acclimation and de-acclimation. Ten one-year-old tea plants were collected from the aforementioned growth chamber and used as non-acclimation controls. The following cold acclimation treatments were applied in this study: CS, cold stimulus, was conducted by exposing SCZ and YH9 plants to 10 °C for 6 h; CA, chilling acclimation, was conducted by exposing SCZ and YH9 plants to low temperature (10 °C day, 4 °C night) for 7 days. Afterwards, FA, freezing acclimation, was conducted by exposing SCZ and YH9 plants to lower temperature (4 °C day, 0 °C night) for another 7 days. Lastly, the plants were exposed to normal temperature (25 °C day, 20 °C night) for 7 days for de-acclimation. After each treatment, the fresh second leaves immediately next to the leaf bud and third leaves downward were collected, and immediately frozen in liquid nitrogen. They were stored at − 80 °C until use. Two independent experiments were conducted for each tea cultivar, and 10 biological replicates were used in each treatment.

### Electrolyte leakage assay and Fv/Fm

SCZ and YH9 were exposed to CA for 1 week, FA for another 1 week and then followed by − 5 °C freezing treatment for 12 h. The tea plants in the control group (NA) were not treated with CA and FA but were exposed to − 5 °C for 12 h. The relative electrolyte leakage rate and chlorophyll fluorescence Fv/Fm value were assayed following methods described previously [26]. The detached leaf discs of SCZ and YH9 were exposed to − 5 °C for 12 h, and Fv/Fm images of leaf discs from NA or FA were obtained with Imaging-Pam chlorophyll fluorescence imager (Walz, Germany).

### RNA extraction and sequencing

The RNAs from collected tissues were extracted using methods described previously [70]. Briefly, the frozen leaves were ground into powder in liquid nitrogen. Then the powder was used for RNA extraction [70]. After preparation, RNA quality and quantity were measured using an agarose gel and the Nanodrop 2500 (Thermo Fisher Scientific, US). RNA-Seq libraries were constructed as described previously [70] and sequenced on the Illumina platform HiSeq X 10. The sequencing format was paired-end 150 bp. The reads are publicly available at the SRA database of NCBI under the project accession number SRP108833.

### Transcript assembly and expression level analysis

All raw reads were cleaned to remove low quality reads as described previously [71], and combined together to build a transcript assembly using the publicly available tea draft genome [29] as the reference sequence, as described previously [71]. Newly discovered genes, that were not annotated in the draft genome, were annotated against publicly available databases using methods described previously [70]. Transcript levels were calculated in FPKM, and differentially expressed genes were defined as exhibiting at least two-fold change in transcript abundance and statistical significance levels of *p* < 0.05 relative to a control by using the tool DESeq2 [31] according to the methods described previously [70].

### Metabolic pathway analysis of DEGs

To analyze DEG-associated functions, we searched the KEGG pathway database using the online tool KAAS (http://www.genome.jp/) [72] based on the Blast bidirectional best hit algorithm with default settings to assign a candidate ko number for each gene. We then mapped the gene to a candidate pathway using KEGG mapper. The sequence databases for *Arabidopsis thaliana* and *Populus trichocarpa* were used as the references. The functional annotation of pertinent DEGs was investigated in the KEGG protein cluster and the UniProt database (http://www.uniprot.org/uniprot/) by protein similarity using the Blastp algorithm with default settings from the corresponding database. DEG functions from two databases were then manually merged. Pathway enrichment analysis was conducted with the tool GOseq [33] according to the methods and scripts described previously [71].

### RT-qPCR validation

Real-time quantitative PCR analyses were conducted with mRNA from the leaves of ten plants from three experimental replicates. The method followed the procedures described in a previous report [26]. The primers for the RT-qPCR are described in Additional file 10: Table S9.

## Additional files

Additional file 1: **Table S1.** Summary of RNA-seq, assembly and annotation. (DOCX 25 kb)

Additional file 2: **Table S2.** Differentially expressed genes and functions in photosynthesis. (DOCX 22 kb)

Additional file 3: **Figure S1.** RT-qPCR validation of selected DEG. (DOCX 452 kb)

Additional file 4: **Table S3.** Differentially expressed genes related to signal transduction in response to cold treatment of SCZ and YH9 leaves. Significant (*P* < 0.05) decrease of transcript abundance is highlighted in blue and bold, significant transcript increase is highlighted in red and bold. (XLSX 22 kb)

Additional file 5: **Table S4.** Differentially expressed transcription factors (TFs) in response to cold treatment of SCZ and YH 9 leaves. Significant (P < 0.05) decrease of transcript abundance is highlighted in blue and bold, significant transcript increase is highlighted in red and bold. (XLSX 28 kb)

Additional file 6: **Table S5.** Differentially expressed cold response genes in response to cold treatment of SCZ and YH9 leaves. Significant (P < 0.05) decrease of transcript abundance is highlighted in blue and bold, significant transcript increase is highlighted in red and bold. (XLSX 21 kb)

Additional file 7: **Table S6.** Differentially expressed genes related to free radical scavengers and flavonoid in response to cold treatment of SCZ and YH9 leaves. Significant (P < 0.05) decrease of transcript abundance is highlighted in blue and bold, significant transcript increase is highlighted in red and bold. (XLSX 19 kb)

Additional file 8: **Table S7.** Differentially expressed genes related to fatty acid in response to cold treatment of SCZ and YH9 leaves. Significant (P < 0.05) decrease of transcript abundance is highlighted in blue and bold, significant transcript increase is highlighted in red and bold. (XLSX 13 kb)

Additional file 9: **Table S8.** Differentially expressed genes related to osmoprotectans in response to cold treatment of SCZ and YH9 leaves. Significant (P < 0.05) decrease of transcript abundance is highlighted in blue and bold, significant transcript increase is highlighted in red and bold. (XLSX 15 kb)

Additional file 10: **Table S9.** Primers used in the RT-qPCR. (DOCX 23 kb)

## Data Availability

The data sets supporting the results of this article are available in the NCBI’s. Sequence Read Archive (SRA) database (accession number: SRP108833. https://www.ncbi.nlm.nih.gov/Traces/study/?acc=SRP108833&go=go).

## Abbreviations

ABA: Abscisic acid
APX: Ascorbate Peroxidase
ARF: Auxin response factor
BAK: Brassinosteroid insensitive 1-associated receptor kinase 1
bHLH: Basic helix-loop-helix protein
BMY: Beta-amylase
CA: a 7-day long-term chill acclimation
CaM: Calmodulin
CBF: C-repeat-binding factors
CBL: Calcineurin B-like Ca^2+^ sensors
CBP: Calcium-binding protein
CDPK: Ca^2+^ − dependent protein kinase
ChiA: Chitinase
CIP: Cold-induced protein
CIPK: CBL-interacting protein kinase-calcineurin B-like protein
CS: a 6 h short-term cold stimulus
CSA: *Camellia sinensis var. assamica*
CSS: *Camellia sinensis var. sinensis*
DA: a 7-day long-term de-acclimation
DEGs: Differentially expressed genes
ERF: Ethylene response factor
FA: a 7-day long-term freezing acclimation
FLS2: LRR receptor-like serine/threonine-protein kinase
Fv/Fm: Chlorophyll fluorescence
GA: Gibberellin
GO: Gene ontology
GR: Glutathione reductase
HSP: Heat shock protein
HXK: Hexokinase
KEGG: Kyoto Encyclopedia of Genes and Genomes
LEA2: Late embryogenesis abundant protein 2
Lhca2: Light-harvesting chlorophyll gene 2
LTP8: Lipid-transfer protein 8
MAPKs: Mitogen-activated protein kinases
MYB86: MYB transcription factor86
NA: No-acclimation
NAC: NAC domain transcription factor
P5CR: Proline-5-carboxylate reductase
P5CS: Proline-5-carboxylate synthetase
PBS: Serine/threonine-protein kinase PBS
PEI: Pectinesterase inhibitors
PRP: Proline-rich protein
REC: Relative electrical conductivity
ROS: Reactive oxygen species
SAD: Stearoy-l ACP desaturase
SCZ: CSS cv. Shuchazao
SnRK2.8: SNF1-related protein kinase 2.8
SOD: Superoxide dismutase
SPS: Sucrose phosphate synthase
SUS: Sucrose synthase
TLP: Thaumatin-like protein
TPS: Trehalose phosphate synthase
WRKY: WRKY transcription factor
YH9: CSA cv. Yinghong9
ZAT: Zinc finger protein
ZEP: Zeaxanthin epoxidase
