# Supplementary material for: Comparative transcriptomic analysis reveals gene expression associated with cold adaptation in the tea plant Camellia sinensis
Source: BMC Genomics. 2019 Jul 31;20:624. doi: 10.1186/s12864-019-5988-3 (PMC6670155; doi:10.1186/s12864-019-5988-3)
Supplement: Supplementary file 1 — Table S1. Summary of RNA-seq, assembly and annotation. (DOCX 25 kb) [file 12864_2019_5988_MOESM1_ESM.docx]

**Additional file 1: Table S1.**  Summary of RNA-seq, assembly and annotation

|  | Sample | Tea cv. SCZ | Tea cv. YH9 |
| --- | --- | --- | --- |
| Transcripts | NA1 | 83,280 | 86,851 |
|  | NA2 | 83,633 | 88,733 |
|  | CS1 | 87,457 | 88,575 |
|  | CS2 | 86,131 | 88,345 |
|  | CA1 | 86,275 | 88,519 |
|  | CA2 | 86,204 | 88,706 |
|  | FA1 | 85,293 | 86,078 |
|  | FA2 | 85,475 | 90,011 |
|  | DA1 | 87,991 | 86,986 |
|  | DA2 | 87,755 | 85,618 |
|  | average | 85,949 | 87,842 |
|  |  |  |  |
| Expressed Genes | NA1 | 45,737 | 46,618 |
|  | NA2 | 45,901 | 47,267 |
|  | CS1 | 46,915 | 47,168 |
|  | CS2 | 46,151 | 47,221 |
|  | CA1 | 45,760 | 46,789 |
|  | CA2 | 45,549 | 46,506 |
|  | FA1 | 45,576 | 45,215 |
|  | FA2 | 45,692 | 46,773 |
|  | DA1 | 47,403 | 47,071 |
|  | DA2 | 47,542 | 46,731 |
|  | average | 46,223 | 46,736 |
|  |  |  |  |
| Mapped Reads | NA1 | 37200531(80.00%) | 34038688(83.29%) |
|  | NA2 | 38803364(83.48%) | 40621681(84.75%) |
|  | CS1 | 37104574(79.41%) | 39031426(85.44%) |
|  | CS2 | 37117040(76.04%) | 41626051(85.63%) |
|  | CA1 | 39038715(80.43%) | 39243914(84.59%) |
|  | CA2 | 39299175(80.62%) | 38866468(85.79%) |
|  | FA1 | 38418454(79.14%) | 35083440(82.58%) |
|  | FA2 | 38216963(80.50%) | 41096345(84.74%) |
|  | DA1 | 39750692(85.23%) | 38752268(80.96%) |
|  | DA2 | 38731824(84.40%) | 36701018(78.00%) |
|  | average | 38368133 (80.93%) | 38506129 (83.58%) |
|  |  |  |  |
|  |  |  |  |
| splice Reads | NA1 | 13881748(29.85%) | 12657739(30.97%) |
|  | NA2 | 14350241(30.87%) | 15276926(31.87%) |
|  | CS1 | 13453093(28.79%) | 13951744(30.54%) |
|  | CS2 | 13380245(27.41%) | 15023446(30.91%) |
|  | CA1 | 14910027(30.72%) | 14349844(30.93%) |
|  | CA2 | 14962851(30.69%) | 14822697(32.72%) |
|  | FA1 | 14316108(29.49%) | 13107990(30.85%) |
|  | FA2 | 14438818(30.42%) | 15182083(31.31%) |
|  | DA1 | 14128912(30.29%) | 14826248(30.97%) |
|  | DA2 | 13733988(29.93%) | 13800120(29.33%) |
|  | average | 14155603 (29.85%) | 14299883 (31.04%) |
