# Supplementary material for: Comparative transcriptomic analysis reveals gene expression associated with cold adaptation in the tea plant Camellia sinensis
Source: BMC Genomics. 2019 Jul 31;20:624. doi: 10.1186/s12864-019-5988-3 (PMC6670155; doi:10.1186/s12864-019-5988-3)
Supplement: Supplementary file 2 — Table S2. Differentially expressed genes and functions in photosynthesis. (DOCX 22 kb) [file 12864_2019_5988_MOESM2_ESM.docx]

**Additional file 2: Table S2.** Differentially expressed genes and functions in photosynthesis

| Gene | Abbreviation | Description | KEGG_ENTRY |
| --- | --- | --- | --- |
| CSA011380 | *Lhca2 | Photosynthesis - antenna protein Lhca2 | K08908 |
| CSA012994 | Lhca4 | Photosynthesis - antenna protein Lhca4 | K08910 |
| CSA024064 | Lhca1 | Photosynthesis - antenna protein Lhca1 | K08907 |
| CSA032834 | Lhca4 | Photosynthesis - antenna protein Lhca4 | K08910 |
| CSA007089 | PsbW | photosystem II reaction center W protein | K02721 |
| CSA009614 | PsbQ | photosystem II reaction center subunit PsbQ | K08901 |
| CSA017077 | psbP | photosystem II reaction center subunit PsbP | K02717 |
| CSA005765 | PetC | cytochrome b6-f complex iron-sulfur subunit 1 | K02636 |
| CSA032248 | *PetF | ferredoxin PetF | K02639 |
| CSA004990 | PsaH-1 | photosystem I reaction center subunit PsaH | K02695 |
| CSA034184 | PsaH-2 | photosystem I reaction center subunit PsaH | K02695 |
| CSA020312 | ATPase a | ATPase a | K02108 |
| CSA004770 | ATPase b | ATPase b | K02109 |
| CSA008691 | ATPase b | ATPase b | K02109 |
| CSA011454 | ATPase b | ATPase b | K02109 |
| CSA013428 | ATPase alpha | ATPase alpha | K02111 |
| CSA015352 | D2 | photosystem II protein D2 | K02706 |
| CSA022661 | PC | photosynthesis electron transport PC | K02638 |
| CSA025110 | FNR | ferredoxin--NADP reductase FNR | K02641 |
