# Supplementary material for: Comparative transcriptomic analysis reveals gene expression associated with cold adaptation in the tea plant Camellia sinensis
Source: BMC Genomics. 2019 Jul 31;20:624. doi: 10.1186/s12864-019-5988-3 (PMC6670155; doi:10.1186/s12864-019-5988-3)
Supplement: Supplementary file 3 — Figure S1. RT-qPCR validation of selected DEG. (DOCX 452 kb) [file 12864_2019_5988_MOESM3_ESM.docx]

[**Additional file 3: Figure S1.**  RT-qPCR validation of selected DEG](#_Toc520661186)

|  |  |
| --- | --- |
|  | |
|  |  |
|  |  |
|  | |
|  |  |
|  |  |
