# Supplementary material for: Comparative transcriptomic analysis reveals gene expression associated with cold adaptation in the tea plant Camellia sinensis
Source: BMC Genomics. 2019 Jul 31;20:624. doi: 10.1186/s12864-019-5988-3 (PMC6670155; doi:10.1186/s12864-019-5988-3)
Supplement: Supplementary file 10 — Table S9. Primers used in the RT-qPCR. (DOCX 23 kb) [file 12864_2019_5988_MOESM10_ESM.docx]

**Additional file 10: Table S9.** Primers used in the RT-qPCR

| Gene ID | Primer Name | Sequence |
| --- | --- | --- |
| LHCA2 | CSA020482--F1 | TCATGGTATGATGCCGGTGC |
|  | CSA020482--R1 | CCCAACATCTGCCTTCGGTT |
| LHCA4 | CSA012994-F1 | TGTCACCGGAATGCTACTGC |
|  | CSA012994-R1 | TCTTGCCACCGTCTGATCTC |
| LHCA4 | CSA032834-F1 | TACGATGCAGGAAAAGCCGA |
|  | CSA032834-R1 | GTGGGTGCAAAATTCAGGGG |
| PsaH-2 | CSA034184-F1 | CCCATGAGATCCACCAGCTA |
|  | CSA034184-R1 | GGGGGTTGTATGGTGAAGGTG |
| D2 | CSA015352-F1 | ACCCAACTCAAGCCGAAGAA |
|  | CSA015352-R1 | GAAGTCATAGGCACGCAGGT |
| ATPa | CSA007724-F1 | GCTTCACCAGGGAAAGAGCA |
|  | CSA007724-R1 | GCGAGCCGACTGGATTTCTT |
| ATPb | CSA004770-F1 | TTGGCTCAGTTGGTTGGAGG |
|  | CSA004770-R1 | CCTCAAGAAACCACCTGCGA |
| ATPb | CSA011454-F1 | TGCCAGGTATTGAGCAGGAAG |
|  | CSA011454-R1 | GCTATCGTGGCATTTCTCGC |
| PsbP | CSA017077-F1 | CGTGTTTACTCAGCACCGAC |
|  | CSA017077-R1 | CCGTCTCTCATTTGCTCCCA |
| FLS2.11 | CSA001565-F1 | CAACAACATCAGCGGCGAAA |
|  | CSA001565-R1 | TAAGCTCGCCATTGAAGCCA |
| PBS1.10 | CSA007045-F1 | TGTTTGAAGTGCTTTGCGGG |
|  | CSA007045-R1 | GGCGAGATTTGGTCCCTCAT |
| PBS1.12 | CSA024279-F1 | TGCGCCTTCAGTACCTTTCC |
|  | CSA024279-R1 | AGTTCCAACAGGACCACACC |
| LHCA1 | CSA003567-F1 | ACCCCTCGCCAAACCTTATC |
|  | CSA003567-R1 | GGGGACGCTTCTCTCCATAC |
